# How to extract and analyze pollen from internal organs and exoskeletons of fossil insects?

**DOI:** 10.1016/j.xpro.2021.100923

**Published:** 2021-10-29

**Authors:** Friðgeir Grímsson, Silvia Ulrich, Reinhard Zetter, Thomas Hörnschemeyer, Michael S. Engel, Sonja Wedmann

**Affiliations:** 1 Department of Botany and Biodiversity Research, University of Vienna, 1030 Vienna, Austria; 2 Department of Paleontology, University of Vienna, 1090 Vienna, Austria; 3 Johann-Friedrich-Blumenbach-Institut für Zoologie & Anthropologie, Georg-August-Universität Göttingen, 37073 Göttingen, Germany; 4 Division of Entomology, Natural History Museum, and Department of Ecology & Evolutionary Biology, University of Kansas, Lawrence, KS 66045, USA; 5 Senckenberg Forschungsstation Grube Messel, Senckenberg Forschungsinstitut und Naturmuseum Frankfurt/M., 64409 Messel, Germany

**Keywords:** Microscopy, Plant sciences, Earth sciences, Evolutionary biology

## Abstract

This protocol explains how to extract pollen from fossil insects with subsequent descriptions of pollen treatment. We also describe how to document morphological and ultrastructural features with light-microscopy and electron microscopy. It enables a taxonomic assignment of pollen that can be used to interpret flower-insect interactions, foraging and feeding behavior of insects, and the paleoenvironment. The protocol is limited by the state of the fossil, the presence/absence of pollen on fossil specimens, and the availability of extant pollen for comparison.

For complete details on the use and execution of this protocol, please refer to Wappler et al. (2015), Ulrich and Grímsson (2020), and Wedmann et al. (2021).

## Highlights

• Protocol for extracting and analyzing pollen grains from fossil insects
• Individual fossil grains can be analyzed using a combined approach
• Simple and fast TEM embedding and sectioning protocol
• Protocol enables a taxonomic assignment of pollen

## Graphical abstract

**Figure:**
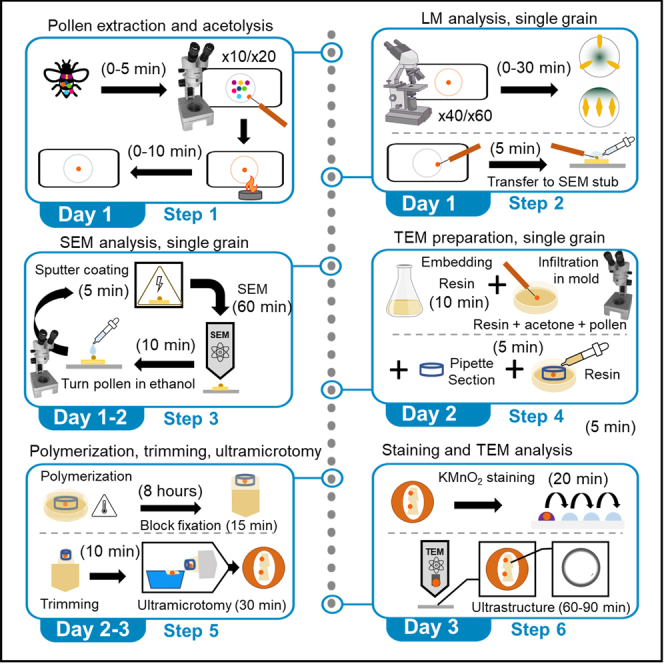

## Before you begin

Make sure to have the desired fossil insect specimens at hand as well as the equipment, tools, and chemicals mentioned/described in the key resources table and in the materials and equipment section.

## Key resources table

**Table:** 

REAGENT or RESOURCE	SOURCE	IDENTIFIER
**Chemicals, peptides, and recombinant proteins**		

Toluidine blue	Sigma-Aldrich	Cat# 6586-04-5, EC Number: 231-760-3
Glycerine, ≥99,5%	Sigma-Aldrich	Cat# 56-81-5, EC Number: 200-289-5
99% acetic anhydride (ReagentPlus, ≥99%)	Sigma-Aldrich	Cat# 108-24-7, EC Number: 203-564-8
96% sulfuric acid	Sigma-Aldrich	Cat# 7664-93-9, Beilstein Registry Number: 2037554
Acetone, ACS reagent, ≥99.5%	Sigma-Aldrich	Cat# 67-64-1, EC Number: 200-662-2, Beilstein Registry Number: 635680
Ethanol (absolute alcohol; ethyl alcohol, pure)	Sigma-Aldrich	Cat# 64-17-5, EC Number: 200-578-6
Formvar solution	Sigma-Aldrich	Cat# 63148-64-1
Xylol	Sigma-Aldrich	Cat# 1330-20-7
Potassium permanganate (KMnO_4_)	Sigma-Aldrich	Cat# 7722-64-7
Agar Low Viscosity Resin Embedding Kit	Agar Scientific Ltd	AGR1078
Spurr Low Viscosity Embedding Kit	Sigma-Aldrich	Product number: EM0300-1KT, RIDADR: UN 3316 9

**Software and algorithms**		

CellSens Standard, XV Image Processing	Olympus	https://www.olympus-lifescience.com/
“analySIS docu” software (Soft Imaging System)	Olympus	https://www.olympus-lifescience.com/

**Other**		

Microscope slides, frosted one side, one end	Sigma-Aldrich	Product Number: CLS294875X25
Micromanipulator (teasing needle with an attached human nasal hair)	Human nose	
Dissecting Needle, straight, plain wooden handle	Avantor	Product number: 25778-000
Gold sputter coater target (Ø 53 × 0,2 mm, disk-shaped, 99,99% Au)	Ögussa, ELECTRON MICROSCOPY SCIENCES	https://www.oegussa.at/de/, https://www.microtonano.com (Product Number: 70-AU5404)
SEM stubs, aluminum	Plano Elektronenmikroskopie	Product Number: G301F
DiATOME Ultra 45° diamond knife (Standard boat, 3 mm cutting edge)	DiATOME	Product number: DU4530
Plastic beaker	Sigma-Aldrich	Product number: Z186848
Forceps (curved tip forceps, with sharp tip)	Sigma-Aldrich	Product number: TR-7-N
Eppendorf microcentrifuge tubes	Sigma-Aldrich	Product number: EP0030122224
Embedding mold (beem flat)	Sigma-Aldrich	Product number: G3654
Loop	Plano-Elektronenmikroskopie	Product number: T5010
Micromanipulator (section manipulators: teasing needle with an attached human eye-lash or deer hair)	Plano-Elektronenmikroskopie	Product number: 119
Lab-oven	Laborhandel24.de	Product number: UN30
Disposable plastic pipettes (Pasteur-Pipettes)	Plano-Elektronenmikroskopie	Product number: G3374-1
Razor blades	Plano-Elektronenmikroskopie	Product number: T585
Two-component epoxy adhesive glue (UHU plus), or cyanoacrylate adhesive (Krazy Glue)	Viking, or Sigma-Aldrich	Product number: SP-AS742742; Z105880
Copper slot grids (3.05 mm, 2×1 mm slot)	Plano Elektronenmikroskopie	Product number: G220T7
Olympus BX50-F light microscope (with a 10× and/or 20× objective lens with approximately 10 mm working distance)	Olympus	https://www.olympus-lifescience.com/
Olympus LMPLFLN10X objective	Olympus	Product number: N2183200
Olympus LMPLFLN20X objective	Olympus	Product number: N2183300
Olympus SZ40 stereomicroscope	Olympus	https://www.olympus-lifescience.com/
Nikon SMZ1270 Stereomicroscope (Epi-fluorescence)	Nikon	https://www.microscopyu.com/techniques/stereomicroscopy/stereomicroscopy-fluorescence-illumination
ColorView IIIu camera (Soft Imaging System)	Olympus	https://www.olympus-lifescience.com/
Jeol JSM-6390 scanning electron microscope	Jeol	https://www.jeolbenelux.com/
Zeiss EM 900 transmission electron microscope	Zeiss	https://www.zeiss.com/microscopy/int/products/scanning-electron-microscopes.html/
Leica EM UC6 ultramicrotome	Leica	https://www.leica-microsystems.com/products/sample-preparation-for-electron-microscopy/ultramicrotomes-cryo-ultramicrotomes/
Sputter Coater Bal-TEC EM SCD 005	Leica	https://www.leica-microsystems.com/products/sample-preparation-for-electron-microscopy/p/leica-em-scd005/

## Materials and equipment

### Acetolysis mixture

**Table:** The acetolysis mixture should be stored in corrosive safety cabinet at 20°C–22°C. The mixture can be stored for about 3 months.

Reagent	Stock concentration	Amount
Acetic anhydride	99%	90 mL (or 9 mL)
Sulfuric acid	95–97%	10 mL (or 1 mL)
**Total**	**n/a**	**100 mL (or 10 mL)**

**CRITICAL:** Fresh made acetolysis mixture (nine to one mix of 99% acetic anhydride and 95–97% sulfuric acid) is highly reactive and reacts intensively with water. The components and the mixture is hazardous (explosive, corrosive) and must be used under a fume hood. After a few days (up to 28 days), the fluid becomes less reactive, more viscous, and turns dark brown, but can still be used for the fast acetolysis method.

### Agar low viscosity resin embedding kit

**Table:** The agar low viscosity resin should be stored at 4°C. The mixture can be stored for maximum 3 days.

Reagent	Final concentration	Amount
Low Viscosity Resin	n/a	6 g
Hardener VH1 ((2-Nonen-1-yl)Succininc anyhrdide)	n/a	3.25 g
Hardener VH2 (Hardener, MNA)	n/a	3.25 g
Accelerator (BDMA)	n/a	0.31 mL (7 drops)
**Total**	**n/a**	**12.5 g**

**CRITICAL:** LV Resin and VH1 hardener are irritant and VH2 hardener and accelerator are corrosive. Although none of the components has the known carcinogenicity of ERL 4206 (Spurr Resin), care should be taken at all stages of handling all resins, and the use of protective gloves and a fume hood or at least a ventilated area is highly recommended.

***Alternatives:*** Spurr Low-Viscosity Embedding Kit (Sigma-Aldrich): D.E.R. 736, Dimethylaminoethanol, ERL 4221, Nonenylsuccinic anhydride.

### Toluidine blue staining

**Table:** The staining solution should be stored at 4°C. The mixture can be stored for up to a year.

Reagent	Final concentration	Amount
Toluidine blue	0.05% w/v	50 mg
Distilled water	n/a	100 mL
**Total**	**n/a**	**100 mL**

Mix the two reagents in a beaker using a magnetic stirrer.

### Potassium permanganate staining

**Table:** The staining agent can be stored at 4°C for an unlimited time.

Reagent	Final concentration	Amount
Potassium Permanganate	1% w/v	1 g
Distilled water	n/a	100 mL
**Total**	**n/a**	**100 mL**

## Step-by-step method details

### Detection and extraction of pollen from fossil insects

**Timing: minutes to hours depending on the number of specimens studied and the number of pollen extracted. To locate and extract a single pollen from a single specimen takes about 5 min.**

This step details how to detect fossil pollen ^1)^ adhering to the exoskeleton of fossil insects or ^2)^ preserved inside the body of fossil insects, and how to extract them so the pollen can be studied with LM, SEM, and TEM.

**Figure 1:**
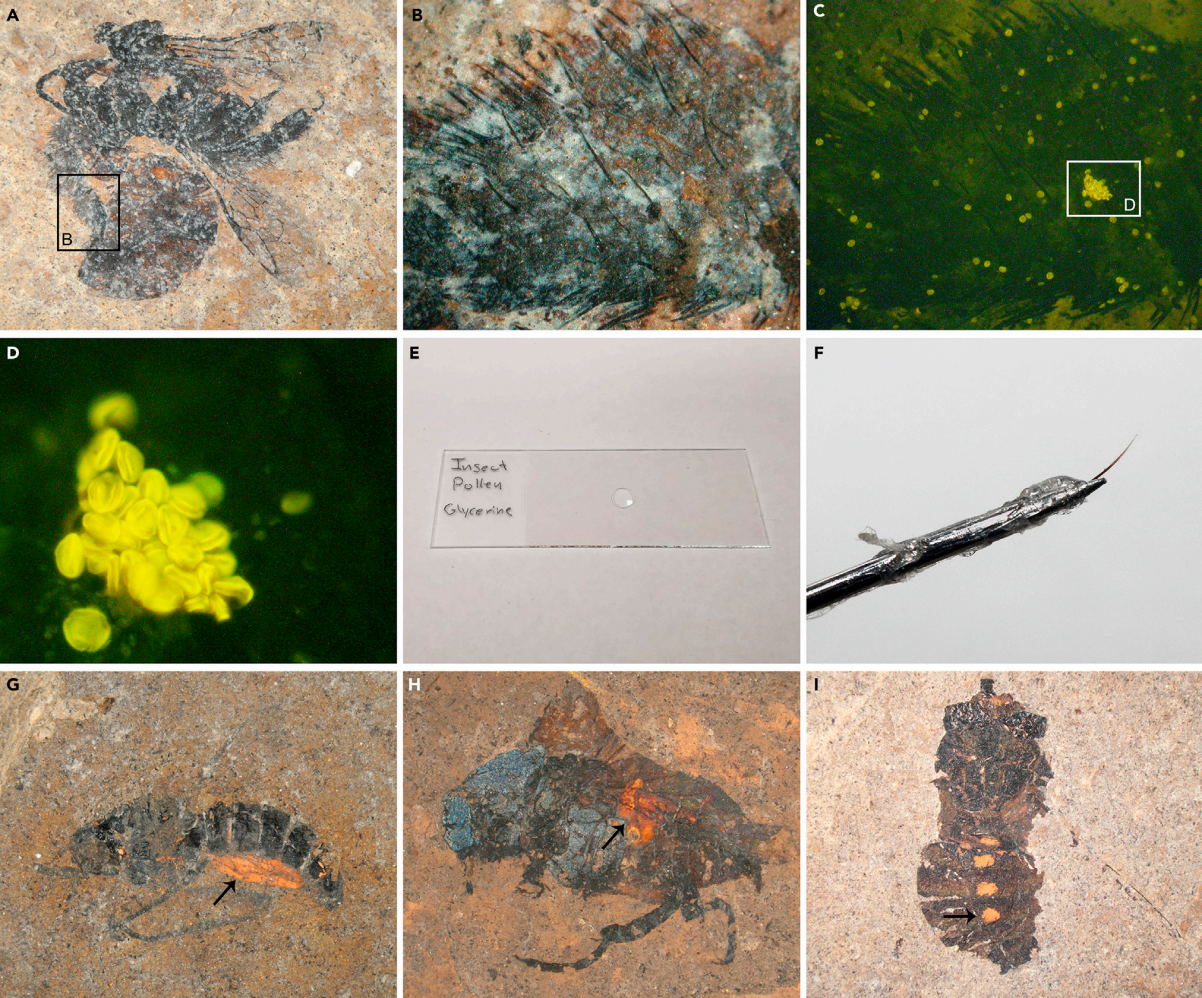
Detection and extraction of pollen from fossil insects (A) Fossil bee observed with stereomicroscope using normal incident light. (B) Close-up of (A) showing part of leg observed with stereomicroscope using normal incident light. (C) Same area as in (B) observed with stereomicroscope using epi-fluorescence illumination. Note how adhering pollen grains illuminate under the epi-fluorescence light source. (D) Close-up of (C) showing a clump of pollen. (E) Drop of glycerine on a LM glass slide. Make the drop small and place it in the middle of the slide. Name the slide appropriately. (F) Micromanipulator, nasal hair attached to a teasing needle with UHU glue. (G) Fossil fly with stomach/abdomen contents preserved (orange; black arrow). Observed with stereomicroscope using normal incident light. (H) Fossil beetle with stomach/abdomen contents preserved (orange; black arrow). Observed with stereomicroscope using normal incident light. (I) Fossil fly observed with stereomicroscope using normal incident light. The thin chitin layer in the gut/abdomen region has been removed in four places with a narrow teasing needle to reveal the otherwise hidden gut contents (yellow; black arrow).
2. To discover pollen adhering to the exoskeleton of a fossil insect turn on the epi-fluorescence illumination. The pollen grains will illuminate when they are hit by the fluorescence beam (Figures 1C and 1D).
3. Place a small drop of glycerine on a LM microscope slide (Figure 1E) and keep it close by.
4. While observing through the stereomicroscope use a teasing needle or micromanipulator (with nasal hair; Figure 1F) to extract pollen from the exoskeleton of the fossil insect.
  a. For dry specimens it is more efficient to dip the needle/micromanipulator into glycerine before you touch the pollen that is adhering to the insect as the pollen grain might otherwise be “blown” away due to electrostatics.
    i. It might be necessary to loosen the pollen from the surface of the insect using a teasing needle and then pick it up with the micromanipulator (nasal hair).
    ii. Push the pollen grain with the wet micromanipulator, tip of the nasal hair, along the surface and it will eventually adhere to the hair.
  b. For wet specimens preserved in glycerine make sure to quickly but carefully dry most of the glycerine from the surface of the insect with a paper napkin.
    i. Take care to let the liquid be absorbed by the tissue and do not scrape the surface of the fossil.
    ii. Then use a teasing needle and/or micromanipulator (nasal hair) to extract the pollen.
5. Transfer and place the pollen into the glycerine drop on the LM microscope slide.

***Note:*** When you dip the tip of the micromanipulator into the glycerine drop, the pollen will automatically detach from the hair and remain in the drop.

1. Place the fossil specimen under the dissecting microscope (stereomicroscope). Adjust the magnification and focus so the insect (Figure 1A) or particular parts of it (leg [Figure 1B], abdomen, head, etc.) are clearly visible.

6. To discover pollen preserved inside the body (digestive system) of fossil insects place the fossil specimen under the dissecting microscope (Figures 1G and 1H).

***Note:*** Check if the abdomen is flat or protruding. Large amounts of pollen within the digestive system of fossil insects are often seen as yellow or orange masses (pointed out by black arrows in Figures 1G and 1H) shining through the thin chitin membrane of a protruding abdomen.

7. Place a small drop of glycerine on a LM microscope slide and keep it close by (Figure 1E).
8. Use a fine teasing needle to puncture the abdomen (Figure 1I) and scrape out part of the gut content.
9. Transfer and place the gut content (pollen) into the glycerine drop on the LM microscope slide (Figure 1E).

***Note:*** When you dip the tip of the micromanipulator into the glycerine drop, the gut content (pollen) will remain in the drop.

### Light microscopy (LM)

**Timing: To transport, stain, and photograph a single pollen grain with LM takes about 25 min.**

This step details how to transport the extracted fossil pollen into staining liquid, prepare it for LM analysis, and how to achieve satisfactory LM micrographs showing important/diagnostic features such as pollen size, polarity, aperture arrangement, P/E ratio, and major pollen wall or structural peculiarities.

10. If the pollen is dirty or showing little contrast (light in color) treat it with acetolysis fluid. If not then skip steps 11 to 16 and continue with step 17.
11. Produce acetolysis solution by mixing nine parts of 99% acetic anhydride and one part 95–97% sulfuric acid.
  a. Fill in 9 mL 99% acetic anhydride into a glass cylinder or bottle under a fume hood.
  b. Add 1 mL 95–97% sulfuric acid using a pipette.
  c. Wait until the fluid has cooled down.

***Note:*** If needed, clean the fume hood after mixing the acetolysis fluid and dispose pipette into hazardous waste. The acetolysis mixture can be stored in a chemical cabinet or fume hood at 20°C–22°C (room temperature).

**Figure 2:**
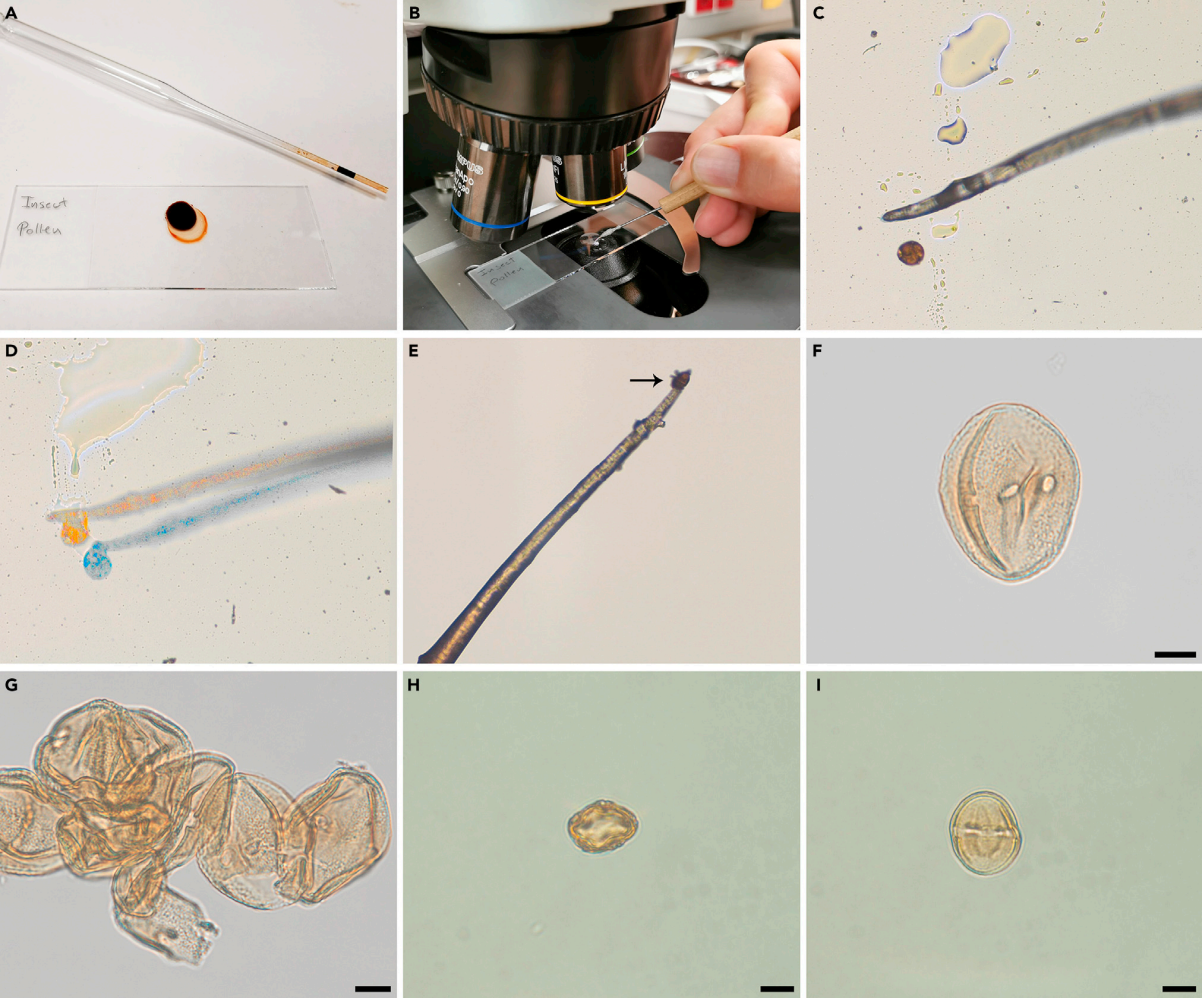
Light microscopy analysis (A) A drop of acetolysis fluid on a LM glass slide. (B) Pollen grain in a drop of glycerine being manipulated using the micromanipulator. Note the c. 10 mm working distance between the sample and the objective lens. (C) Pollen grain being pushed out of a glycerine drop using the tip of a micromanipulator (nasal hair). (D) Pollen grain being picked up from a LM glass slide with a micromanipulator. (E) Pollen grain (black arrow) seen adhering to the tip of a micromanipulator. (F) Example of a single pollen grain (*Parthenocissus*) from the gut/abdomen of a fossil fly photographed in equatorial view with LM. Scale bar: 10 μm. (G) Example of a pollen clump from the gut/abdomen of a fossil fly photographed with LM. Scale bar: 10 μm. (H) Example of a single pollen grain (Sapotaceae) from the gut/abdomen of a fossil fly photographed in polar view (optical cross section) with LM. Scale bar: 10 μm. (I) Same grain as in (H) in equatorial view. Scale bar: 10 μm.
13. Observe the fossil pollen grain in the glycerine drop on the old LM microscope slide with an erect image compound microscope equipped with a 10× or 20× objective lens having an approximately 10 mm working distance (Figure 2B).
14. While observing the pollen grain through the eyepiece of the LM move the distal end of the micromanipulator (with nasal hair) between the LM microscope slide and the lens.
  a. Gently dip the tip of the micromanipulator into the glycerine and use it to pick up the pollen grain stored within the glycerine drop.
  b. Transfer the pollen grain to the acetolysis liquid on the new LM microscope slide.

***Note:*** To extract the pollen grain from the glycerine drop, brush, or push the pollen grain towards the edge of the glycerine using the tip of the micromanipulator, then push it out of the glycerine until it adheres to the nasal hair (Figures 2C–2E) and transfer to the acetolysis drop.

***Note:*** When you dip the tip of the micromanipulator with the attached pollen into the acetolysis liquid the pollen will automatically detach from the hair and remain in the drop.

12. Place a drop or two of acetolysis fluid on a new LM microscope slide (Figure 2A) and place the slide on a paper sheet under a fume hood.

15. Light a tea candle and hold the LM microscope slide, with the pollen grain in the acetolysis drop, over the candle flame for 3–5 s to dissolve extra organic material on the surface of the pollen grain, to “rehydrate” the pollen grain if possible, and to stain the pollen grain for LM photography.

***Note:*** Do not hold the LM microscope slide over the flame for too long or the pollen grain will become too dark for optimal photography. Best is to heat the slide shortly (3–5 seconds) and then place it under the LM to check the status of the pollen grain. This should be repeated until the pollen grain has gained the required color.

16. Place the LM microscope slide with the pollen grain in the acetolysis drop under the erect image compound microscope. Use the micromanipulator to extract the pollen grain from the acetolysis drop and transfer it back to the glycerine drop on the old LM microscope slide.
17. Observe the pollen grain in the glycerine drop with an erect image compound microscope equipped with a 10× or 20× objective lens having an approximately 10 mm working distance (Figure 2B).
18. Use the micromanipulator to turn the pollen to an optimal position (polar and equatorial views) while observing it through the eyepiece of the LM.
  a. When the pollen grain has been placed in an optimal position switch to a lens with higher magnification (50× or 60×).
  b. Photograph the pollen grain at different foci (high-, low focus, optical section; see Halbritter et al., 2018, p. 85–95) (Figures 2F–2I).

***Note:*** Make sure to document important LM features such as size, outline in polar and equatorial views, sculpture, aperture arrangement, and irregularities in the pollen wall structure.

### Scanning electron microscopy (SEM)

**Timing: To transport, wash, sputter coat, and photograph a single pollen grain with SEM takes about 2 h.**

This step details how to transport the pollen following the LM analysis onto SEM specimen stubs, how to clean the pollen and prepare for SEM, and how to achieve satisfactory SEM micrographs showing important/diagnostic sculpture types and other features of the pollen surface.

**Figure 3:**
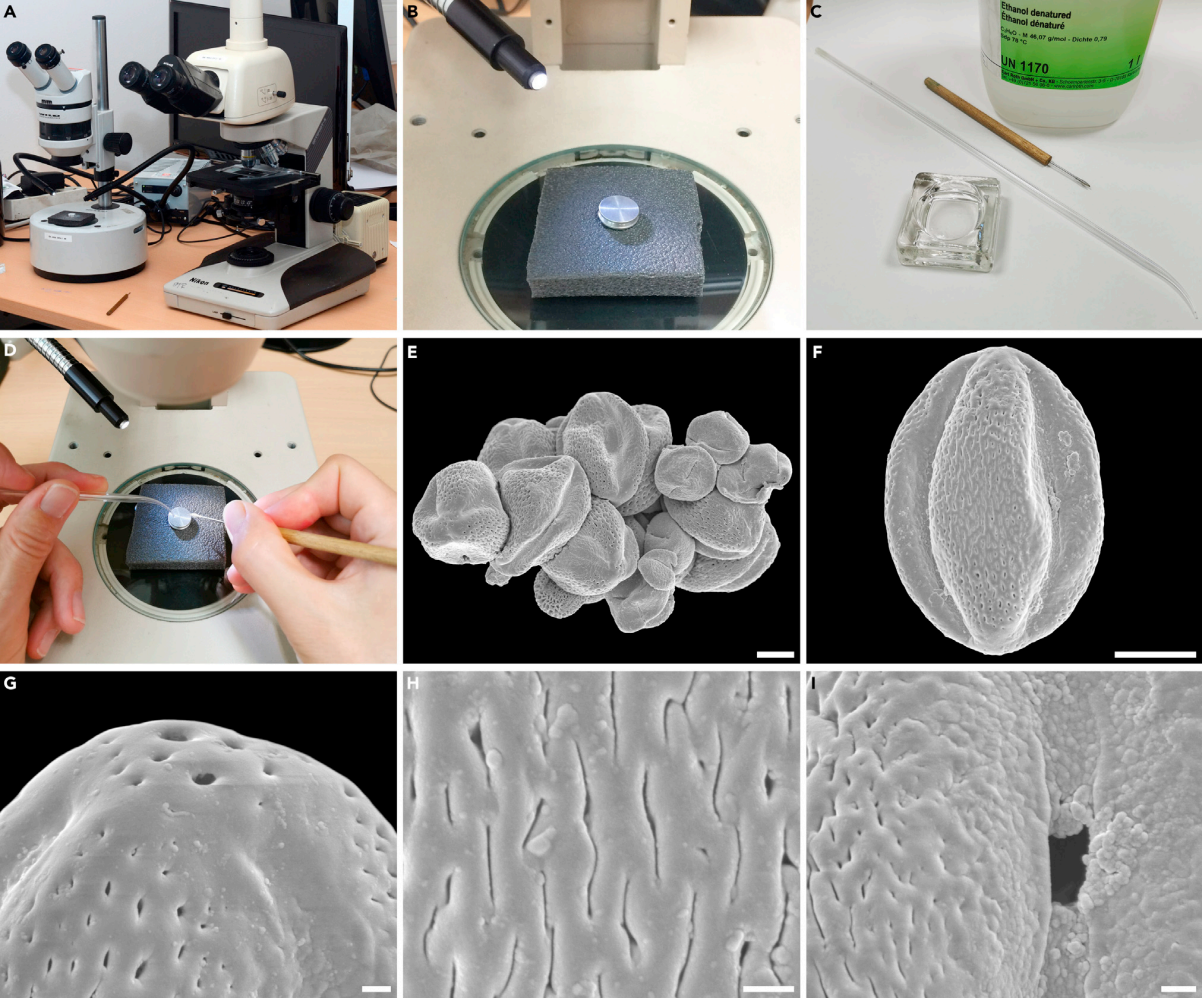
Scanning electron microscopy analysis (A) Binocular stereomicroscope (left) close beside an erect image compound microscope (right) equipped with a 10× lens having a c. 10 mm working distance. (B) SEM specimen stub positioned under a stereomicroscope using normal incident light (from the side). (C) Ethanol, small glass container, micromanipulator, and a glass pipette with a narrow and pointed end to transport ethanol. (D) Use both hands simultaneously, form a drop on the surface of the SEM specimen stub with the left and then dip the tip of the nasal hair with the fossil pollen into the drop with the right. (E) Clump of pollen from the abdomen of a fossil fly observed with SEM. Scale bar: 10 μm. (F) Example of an overview micrograph showing a single pollen grain (*Parthenocissus*) from the abdomen of a fossil fly observed with SEM. Scale bar: 10 μm. (G) Example of a close-up micrograph showing polar area of a pollen (*Parthenocissus*) grain from the abdomen of a fossil fly observed with SEM. Scale bar: 1 μm. (H) Example of a close-up micrograph interapertural area of a pollen (*Parthenocissus*) grain from the abdomen of a fossil fly observed with SEM. Scale bar: 1 μm. (I) Example of a close-up micrograph showing aperture region of a pollen (*Parthenocissus*) grain from the abdomen of a fossil fly observed with SEM. Scale bar: 1 μm.
20. Place a single SEM specimen stub under the stereomicroscope (Figure 3B) and have a small container with fresh ethanol at your side as well as a narrow pointed glass-pipette (Figure 3C; for how to make such pipettes from 2 mm wide glass pipes consult Halbritter et al. (2018, p. 121–122).
21. Locate the pollen grain in the glycerine drop on the old LM microscope slide with an erect image compound microscope.
22. While observing the pollen grain through the eyepiece of the LM move the distal end of the micromanipulator (with nasal hair) in-between the LM microscope slide and the lens and then gently dip the tip of the micromanipulator into the glycerine.
  a. Use the micromanipulator to pick up the pollen grain stored within the glycerine drop.
  b. Slowly move it towards the stereomicroscope.
23. Dip the tip of the narrow pointed glass-pipette into the ethanol container and it automatically sucks up a small portion of ethanol.
  a. Press the tip of the pipette on the surface of the SEM specimen stub to leave a tiny drop of ethanol on the SEM specimen stub.
  b. Gently press the tip of the micromanipulator, with the attached pollen, into the drop of ethanol and the pollen will detach from the hair, float a bit in the ethanol drop, and finally rest on the surface of the SEM specimen stub when the ethanol has evaporated (Figure 3D).

***Note:*** Try to make the ethanol drop small and close to the center of the SEM specimen stub. Two to three additional ethanol drops should be added to clean the glycerine thoroughly off the pollen grain (glycerine is miscible in ethanol).

***Note:*** If pollen grains are sputter coated without being completely cleared of glycerine their sculpture will be obscured by the glycerine and cannot be studied properly with SEM.

19. Position a stereomicroscope close beside an erect image compound microscope equipped with a 10× or 20× objective lens having an approximately 10 mm working distance (Figure 3A).

24. Sputter coat the material placed on the SEM specimen stub with gold for 5 min and a gold coating thickness of 20 nm and photograph the pollen using a SEM (overview and close-ups) (Figures 3E–3I).

***Note:*** Pollen of particular interest can be turned and photographed from different sides. To do this, place the SEM specimen stub under the stereomicroscope again. While observing the pollen grain through the eyepiece add a drop of ethanol to the sputtered sample so it engulfs the pollen grain and then flip the grain over using the micromanipulator before the ethanol evaporates. Then re-sputter the SEM specimen stub with the pollen grain and photograph it again using the SEM. This applies especially to any kind of heteropolar pollen/spores or tetrads of some sort.

### Transmission electron microscopy (TEM)

**Timing: To transport, wash, embed, stain, cut, and photograph a single pollen grain with TEM takes about 2 days.**

This step details how to transport the pollen following the SEM analysis into glycerine, how to clean and dehydrate the pollen, how to embed and stain it, how to cut it into sections, and how to achieve satisfactory TEM micrographs showing important/diagnostic ultrastructural features in different areas of the pollen wall.

**Figure 4:**
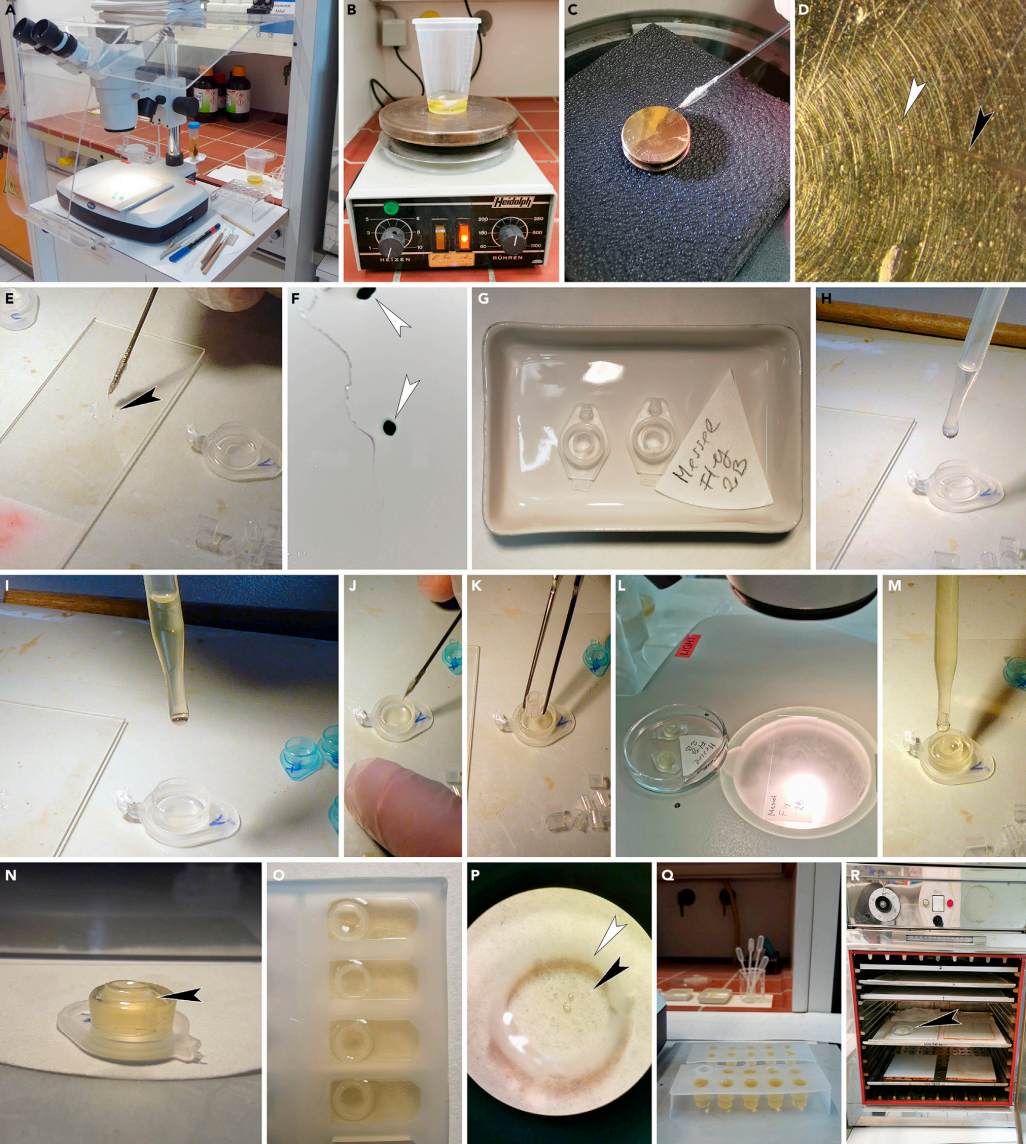
Transmission electron microscopy: Infiltration and embedding (A) Setup for TEM preparation with stereomicroscope inside a fume hood. (B) Mixing the four components of the embedding solution. (C) Transfer pollen from the SEM specimen stub under a stereomicroscope. (D) Close-up of (C) showing pollen picked up (white arrowhead) from the SEM specimen stub using a glycerine-soaked micromanipulator (black arrowhead). (E) Drop of glycerine on a LM microscope slide with transferred gold-coated pollen inside (arrowhead). (F) Close-up showing the extracted gold-coated pollen grain in glycerine (white arrowheads). (G) Example of a clean embedding mold with flat inner surface (e.g., Eppendorf lids). In each embedding mold only one single pollen grain, or a whole pollen clump, is placed. (H) Acetone is used dropwise to clean the pollen grain from glycerine inside the embedding mold. (I) For infiltration the embedding media is added to the acetone into the embedding mold. (J) Micromanipulator is used to move/keep the pollen grain in the center of the embedding mold. (K) The plastic pipette section is used to restrain the pollen grain in the center of the embedding mold. (L) Infiltration inside the embedding mold takes as long as the acetone needs to evaporate. This can be observed under the binocular stereomicroscope, as the pollen grain is floating inside the acetone-embedding mixture as long as acetone is still present. (M) Mold filled up with embedding media. (N) Example showing how to fill up the embedding mold (arrowhead), without overfilling it. (O) Example showing another type of a flat embedding mold. (P) Close-up of (O) showing pollen grain (black arrowhead) inside plastic pipette section (white arrowhead). (Q) Block holders of different sizes, made in embedding molds with remaining embedding media. (R) Polymerization of the embedded fossil pollen grain(s) inside the oven at 70°C.

25. Before the pollen grain can be transferred from the LM microscope slide (or the SEM specimen stub), the setup for TEM infiltration and embedding must be prepared (Figure 4A).
  a. Prepare the embedding solution (LV-Resin). Mix the components in a clean (dust-free) disposable lockable plastic beaker using a magnetic stirrer (Figure 4B).
  b. The first two components must be mixed before adding the remaining ingredients, then mix well again.
  c. The mixture can be used immediately for infiltration and embedding. Inside the closed beaker, the embedding solution can be stored in a refrigerator at 4°C for 3 days.
    ***Note:*** Agar low-viscosity resin (LV-resin; Agar Scientific, 2004) and Spurr’s low-viscosity epoxy resin (Spurr, 1969) are suitable embedding media providing complete and uniform penetration of fossil pollen grains.
  d. Place a stereomicroscope, the embedding solution (LV-Resin), and a plastic pipette inside a fume hood.
  e. Place an embedding mold, with a flat/smooth inner surface, under the binocular. Turn hood settings on lowest intensity in order not to lose pollen grains due to strong airflow.
  f. Clean the embedding mold before embedding the pollen grain as extra particles might interfere with the embedding material: Add pure acetone dropwise into the empty mold and use a pipette to reabsorb and dispose of liquids. Repeat this step until the embedding mold is clean.

26. Place the SEM specimen stub with the coated pollen under a stereomicroscope (Figure 4C).
27. Dip the micromanipulator (with nasal hair) into glycerine and then use it to pick up the pollen grain from the SEM specimen stub (Figure 4D).

***Note:*** When the glycerine-soaked micromanipulator is brushed against the pollen, laying on the SEM specimen stub, the pollen grain will adhere to it.

28. Transfer the pollen grain into a drop of glycerine placed on a new LM microscope slide and study it with the LM to make sure you have extracted the correct particle (Figure 4E). The pollen grain will look black and non-transparent due to the gold-coating (Figure 4F).

***Note:*** The pollen grain can also be transferred directly from the SEM specimen stub into the embedding mold.

29. Use the micromanipulator to transfer the pollen grain into the embedding mold (Figure 4G).

***Note:*** Striking the micromanipulator along the bottom of the embedding mold will transfer the pollen grain into the mold where it will remain (glycerine makes it sticky).

30. To clean the pollen grain, add acetone dropwise into the embedding mold until the mold is half-full (Figure 4H).
31. For infiltration add a few drops of fresh embedding media (1:2, acetone/LV-resin) into the embedding mold (Figure 4I).

***Note:*** Use the micromanipulator to move the pollen grain towards the center of the embedding mold (Figure 4J).

32. To prevent pollen grains from adhering to the wall of the embedding mold, a small section from a plastic pipette (1 mL pipette, 5 mm in diameter) is used to restrain the pollen grain in the center of the embedding mold (Figure 4K). Use forceps to place the pipette section into the embedding mold and over the pollen grain.

***Note:*** The plastic section is soft and easily trimmed with a razor blade.

33. Again, use the micromanipulator to move the pollen grain towards the center of the embedding mold.
34. Let the sample stand for approximately 15 min at 20°C–22°C (room temperature) until the acetone has evaporated (Figure 4L).
35. Dropwise fill up the embedding mold with embedding media (Figures 4M–4O).

***Note:*** Do not overfill the mold since that might cause the plastic pipette section to float and the suspended pollen grain might get lost (Figure 4N).

36. If needed, use the micromanipulator to move and manipulate suspended pollen grains inside the resin towards the center (Figure 4P).
37. Remaining embedding media can be used to make block holders, and can be filled up in bigger embedding molds (Figure 4Q).
38. Polymerize the sample and the block holders at 70°C for 6–12 h (Figure 4R).

***Note:*** Polymerization time varies with the amount and type of resin.

**Figure 5:**
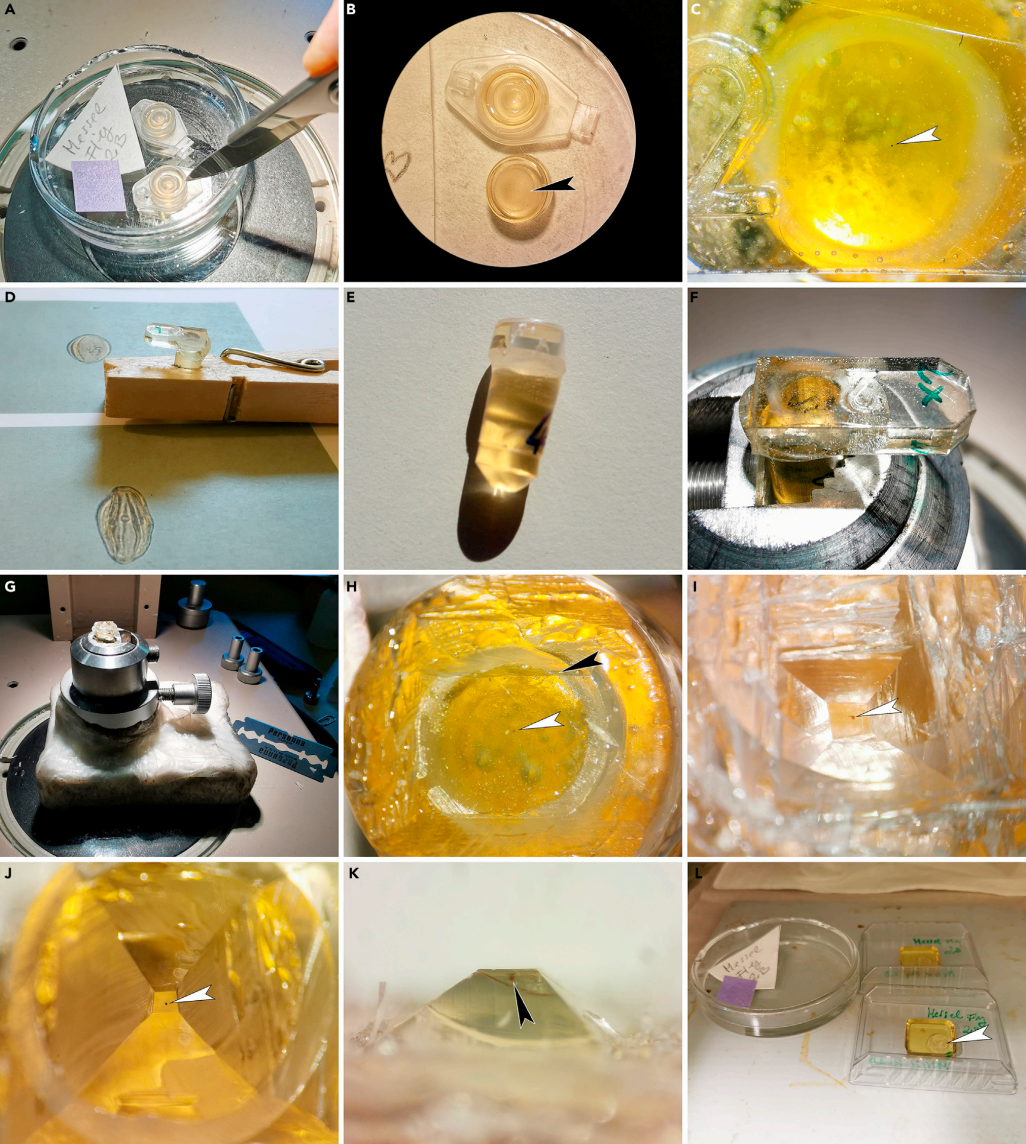
Transmission electron microscopy: Trimming (A) Cutting out the specimen block from the disposable embedding mold. (B) The pollen grain is positioned close to the flattened distal side of the specimen block (arrowhead). (C) Under the binocular the pollen grain can be located inside the specimen block (arrowhead). (D) Specimen block fixed with two-component epoxy adhesive glue to a pre-made block. (E) Example of a ready-made specimen block. (F) Example of a ready-made specimen block fixed in a block holder. (G) Trimming the specimen block under the binocular using razor blades. (H) The soft plastic section can be easily trimmed with a razor blade (black arrowhead). The pollen grain (white arrowhead) is observable in the center of the section. (I) Example of a trimmed specimen block, with a trapezoid form, and a final block face between 2 and 4 mm^2^. The uncoated pollen grain is ideally positioned in the center (arrowhead). (J) Example of a trimmed specimen block with a single gold-coated pollen grain inside (arrowhead). (K) Specimen block seen from a trimmed side, with the pollen grain located in appropriate level (arrowhead) for sectioning. (L) Example of a re-embedded specimen block, with the cut-out part of the specimen block including the pollen turned in the desired position (arrowhead).

***Note:*** The pollen grain should now be observable with a binocular and positioned close to the flattened distal side of the specimen block (Figures 5B and 5C).

39. Cut the specimen block out from the disposable embedding mold using a razor blade or scalpel (Figure 5A).

40. Fix the specimen block to a larger and round pre-made block (or any other type of block holder; see Embedding Molds in Zavialova et al., 2018, pages 123–125), using a two-component epoxy adhesive glue (Figure 5D; UHU plus for bigger blocks) or a cyanoacrylate adhesive (Krazy Glue for smaller blocks).
41. The specimen block is now trimmed sidewise into a trapezoid form using razor blades (Figures 5E–5K). The size of the final block face should be between 2 and 4 mm^2^.
42. The specimen block should now be ready for ultrathin sectioning.

***Note:*** If the orientation of the pollen grain needs to be adjusted, the part of the specimen block including the pollen is cut off, turned in the desired position, and re-embedded (Figure 5L).

**Figure 6:**
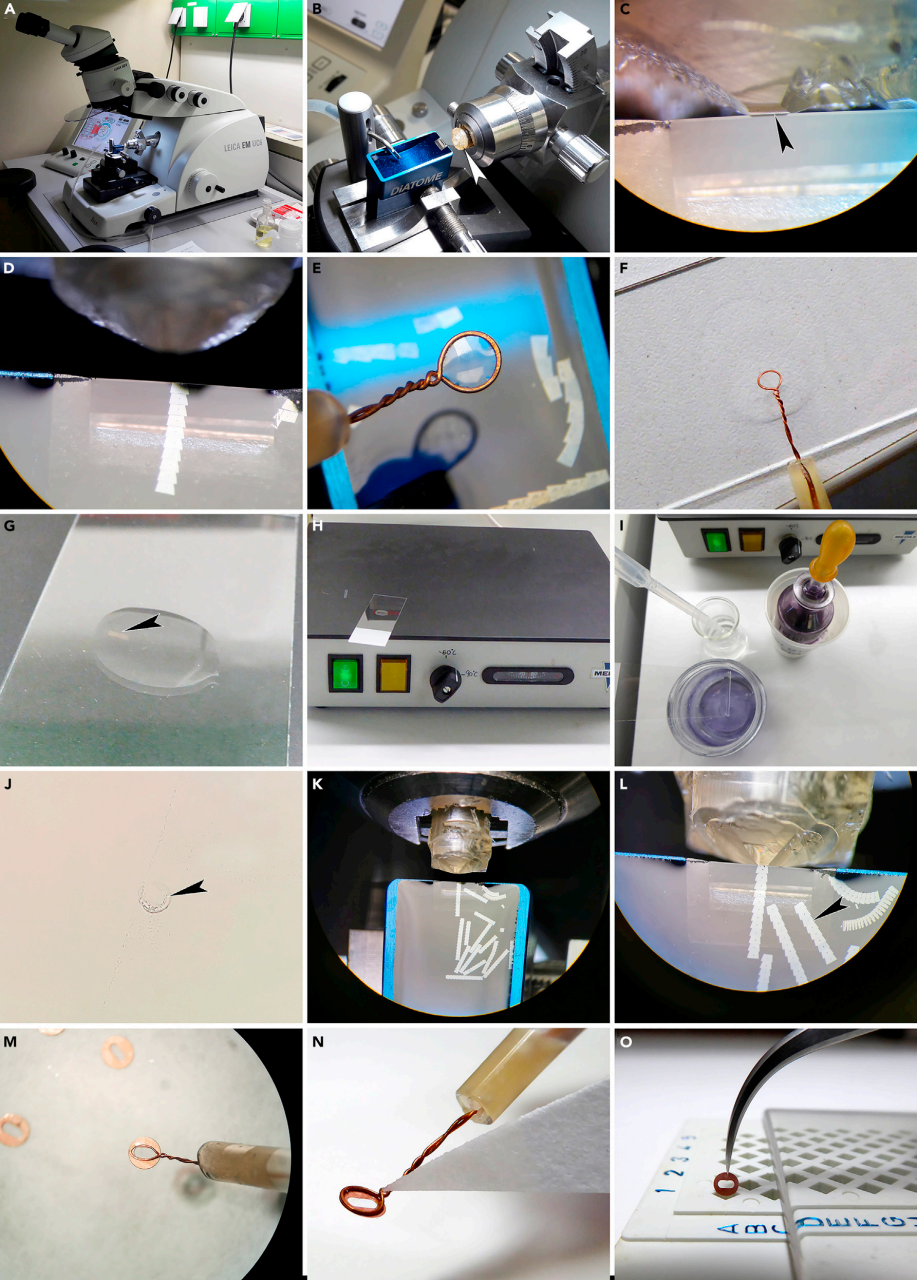
Transmission electron microscopy: Ultramicrotomy (A) Ultramicrotome for ultrathin sectioning. (B) Specimen block (arrowhead) with block holder adjusted in the specimen-holding arm of the ultramicrotome. (C) Block adjustment parallel to the knife-edge (arrowhead). (D) Ultrathin sections (50–70 nm, silver to light gold) floating on the water surface. (E) Picking up sections with a loop. (F) The first ultrathin sections are transferred into a drop of water on a glass slide, to establish when the pollen grain has been reached. (G) Transferred sections floating on water drop (arrowhead) are visible by the reflected (interference) light. (H) Slide placed on hot plate to speed up the drying process. (I) Dry section(s) on the glass slide are stained with toluidine blue for LM analysis. (J) Example of an ultrathin section stained with toluidine blue under the LM. The cross section of the pollen wall (black arrowhead) of a fossil pollen grain is now clearly visible in LM with a 10× or 20× objective lens. (K) Example of a single fossil pollen grains cut as a whole, in a series of ultrathin sections (70 nm). (L) For picking up sections with the loop, the floating section-row(s) are separated into smaller groups (arrowhead) using two micromanipulators (eyelash). (M) The stretched sections are transferred onto formvar film-coated copper slot while observing with a stereomicroscope. (N) Water is removed slowly using the tip of a pre-cut filter paper. (O) Grids are stored within a box with the sections facing all the same direction.

***Note:*** Since the pollen grain is already positioned at the appropriate level for sectioning, semi-thin sections from the tip of the block face are not necessary.

43. The specimen block can now be fixed in a block holder (Figure 5F) and adjusted in the specimen-holding arm of the ultramicrotome (Figures 6A and 6B).

44. The surface of the specimen block is already flat and therefore ultra-thin sections can now be produced using a DiATOME Ultra 45° diamond knife.
  a. The knife is placed in the knife holder and the knife boat filled with distilled water.
45. The block has to be adjusted as precise as possible to the knife using the coarse and fine advance capabilities.
  a. The block is adjusted parallel to the knife-edge by using the reflecting light (Figure 6C).
46. The cutting window is now adjusted before starting the cutting process.
47. Ultrathin sections (70 nm, Figure 6D) are cut with a slow cutting stroke (cutting speed of 1mm/s) and slow return speed.
48. To establish if the pollen grain has been reached, when contrasting structures appear in the ultrathin sections, a section is picked up for observation with LM (Figures 6E–6J).
  a. A single section is isolated using micromanipulators (eyelashes), picked up with a loop, and transferred into a drop of water on a glass slide (Figures 6E–6G).
  b. The slide is placed on a hot plate (70°C; Figure 6H). The dry section is then stained with toluidine blue for a few seconds (Figure 6I), washed with distilled water, and viewed under LM (Figure 6J).
  c. If the pollen grain has been reached, the cutting process can continue.
49. The single fossil pollen grains can be cut as a whole (Figure 6K), in a series of ultrathin sections (70 nm).
50. Separate the floating section-row(s) into smaller groups (Figure 6L) using two micromanipulators (eyelash) so the section groups can be picked up with a loop.

***Note:*** Depending on the size, a group can contain as many sections as fit within the loop.

51. Stretch the sections before picking them up using xylol. Use a small piece of filter paper moistened with a drop of xylol and move it closely above the sections floating on the water surface.

***Note:*** The xylol vapors stretch the sections and they become thinner.

52. Use a loop to transfer the stretched ultra-thin sections and place them onto a formvar film-coated copper slot grid while observing with a stereomicroscope (Figure 6M).
53. Slowly remove water from the loop using the tip of a pre-cut wedge shaped filter paper touching the twisted end of the loop (Figure 6N).
54. Transfer the grid(s) using curved tip forceps into a grid-box. Make sure that all grids are oriented in the same direction within the box so all the sections are facing the same direction (Figure 6O).
  a. Write a sectioning protocol, including specimen details and grid-box number.
  b. Store the box upside down or away from light to protect the sections from light.
55. For staining, treat the ultra-thin sections on the copper grids with 1% aqueous potassium permanganate solution (Figures 7A–7C).
  a. Place small drop(s) of potassium permanganate solution, using a pipette, on a piece of parafilm (Figure 7A).
  b. Use forceps to transfer the grid(s) from the box and place them with the section-side directed downwards onto the staining drop(s). Stain the sections for 5–7 min.
  c. Make a row of 3–5 large drops of distilled water on a piece of parafilm for washing (Figure 7B).
  d. Transfer the grids from the staining agent using the forceps and wash each grid three times for 5 min in the water drops (Figure 7C).
  e. Put the grids back into the grid box using the forceps and use a piece of filter paper to soak water from the grids.
  f. The ultra-thin sections are now ready for TEM analysis.

**Figure 7:**
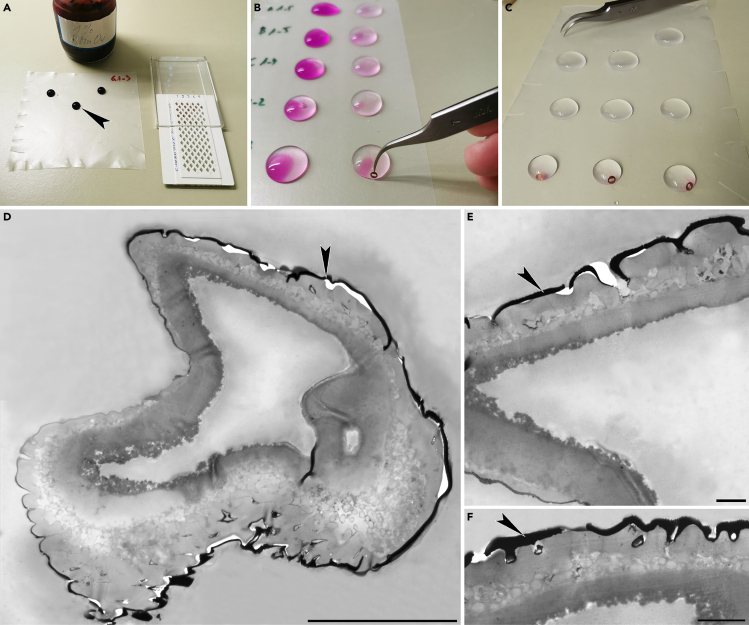
Transmission electron microscopy: Staining and analysis (A) The ultra-thin sections on copper grids are stained in drop(s) of 1% aqueous potassium permanganate solution (black arrowhead) on a piece of parafilm. (B) The staining solution is washed out in large drops of distilled water. The forceps are used to transfer the grid(s) and place them with the section-side directed downwards onto the drop(s). (C) Rows of water drops showing the grids floating on the water surface. (D) Example of an overview micrograph showing the cross section of a single pollen grain (*Parthenocissus*) from the abdomen of a fossil fly observed with TEM. Note the electron-dense gold-coating (arrowhead). Scale bar: 10 μm. (E) Example of a close-up micrograph in interapertural area of a pollen (*Parthenocissus*) wall from the abdomen of a fossil fly observed with TEM. Note the electron-dense gold-coating (arrowhead). Scale bar: 1 μm. (F) Example of a close-up micrograph in interapertural area of a pollen (*Parthenocissus*) wall from the abdomen of a fossil fly observed with TEM. Note the electron-dense gold-coating (arrowhead). Scale bar: 1 μm.
56. Study the ultrastructure of the pollen wall with TEM. Make sure to photograph both interapertural (Figures 7E and 7F) as well as apertural areas. Note all pollen wall peculiarities.

## Expected outcomes

Previous work on pollen grains extracted from fossil insects (e.g., Krassilov and Rasnitsyn, 1997; Krassilov et al., 1999, 2003, 2007; Tekleva 2015) have not provided any detailed accounts on how to observe, extract, or investigate the pollen. This protocol provides clear written instructions and supportive illustrations enabling scientists to successfully extract and analyze fossil pollen from the exoskeleton or internal organs of fossil insects. When the pollen grains have been studied via the combined LM, SEM, and TEM protocol presented herein, their morphological and ultrastructural characteristics can be used to systematically place the pollen and make conclusions about their paleovegetational, paleophytogeographical, paleoecological (flower–insect interactions), and paleoclimatological significance.

## Limitations

The method is limited by the state of the fossil and the presence/absence of pollen on fossil specimens. Not all fossil insects will have adhering fossil pollen. Also, the preservation and the mineral combination might affect the possibility of extracting pollen from the exoskeleton of fossil insects. It might complicate the extraction of the pollen and more force would be needed to loosen the microfossils from the insect. Using teasing needles for this purpose might leave permanent scars in the fossil specimen. If the gut contents of fossil insects are preserved or not may not always be detectable via observation with a stereomicroscope, even when using epi-fluorescence illumination, since the chitin layer might conceal the gut contents. If the stomach/abdomen region of a fossil insect looks as though it is protruding or relatively swollen, it might be worthwhile to puncture it with a teasing needle and scrape out some material to check for pollen. Again, this will leave a permanent scar in the fossil specimen.

## Troubleshooting

### Problem 1

There is no pollen observed on/inside the fossil insect (step 2).

### Potential solution

Not all fossil insects will have adhering pollen, it is an uncommon occurrence. Replace the fossil insect with another specimen and focus on groups that are presently known to come into contact with pollen, incl. bees, wasps, butterflies, beetles, and flies.

### Problem 2

The pollen looks dirty or the surface of the grain seems to be covered by foreign particles (step 17).

### Potential solution

Some organic particles can be hard to remove without physical force. After acetolysis, push the pollen grain towards the margin of the glycerine drop and use the hair to press on the pollen grain while rubbing back and forth and scrape off the foreign particles.

### Problem 3

The mold is overfilled with resin, the plastic pipette section is floating, and the pollen grains are out of sight (step 35).

### Potential solution

Locate the pollen, wait until it sinks towards the bottom, then use a micro-pipette to carefully suck away extra resin from the embedding mold. Remove resin until the plastic pipette section settles. Use the micromanipulator (nasal-hair) to transport the pollen into the center of the plastic pipette section.

### Problem 4

The resin is not hardening within the given time period (step 38).

### Potential solution

The polymerization period must be prolonged. The resin might not be properly mixed, or the amount of accelerator was insufficient. Keep the resin inside the oven until it is completely polymerized (up to a few days, if needed). If the resin is not polymerizing at all, the chemical components used might have been flawed. Mix a new embedding medium with fresh components and transfer the fossil pollen into the new resin.

### Problem 5

When the specimen block including the pollen is re-embedded the pollen grain is no longer positioned at the appropriate level for sectioning (step 42)

### Potential solution

To reach the pollen grain within the specimen block, trimming must be conducted prior to semi-thin sectioning. Trimming is repeated as in step 41, but also the tip of the pyramid is cut away until the appropriate level within the sample has been reached. Trim close to the pollen grain but make sure not to cut into it. Continue trimming using the ultramicrotome.

## Resource availability

### Lead contact

Further information and requests for resources and reagents should be directed to and will be fulfilled by the lead contact, Friðgeir Grímsson (fridgeir.grimsson@univie.ac.at).

### Materials availability

This study did not generate new or unique reagents

## Data Availability

This study did not generate/analyze any datasets or codes.
